# Association between patient adherence and treat-to-target in gout: A cross-sectional study

**DOI:** 10.1097/MD.0000000000037228

**Published:** 2024-02-23

**Authors:** Shasha Hu, Sihui He, Jianyong Zhang, Wukai Ma, Hongling Geng, Zhiying Zhan, Xueming Yao, Li Zhong, Jiaxin Wei, Xia Qiu, Ertao Jia

**Affiliations:** a The Department of Rheumatology, Shenzhen Traditional Chinese Medicine Hospital, Shenzhen, China; b Shenzhen Traditional Chinese Medicine Hospital Affiliated to Nanjing University of Chinese Medicine, Shenzhen, China; c The Department of Rheumatology, the Fourth Clinical Medical College of Guangzhou University of Chinese Medicine, Shenzhen, China; d The Department of Rheumatology, the Second Affiliated Hospital of Guizhou University of Traditional Chinese Medicine, Guiyang, China; e The Department of Gynecology, Guangdong Provincial Hospital of Chinese Medicine, the Second Affiliated Hospital of Guangzhou University of Chinese Medicine, Guiyang, China; f Department of Epidemiology and Health Statistics, Fujian Provincial Key Laboratory of Environment Factors and Caner, School of Public Health, Fujian Medical University, Fuzhou, China.

**Keywords:** gout, medication adherence, treatment

## Abstract

The implementation of a treat-to-target (T2T) approach has been widely recommended for achieving optimal outcomes in gout treatment, as substantiated by a wealth of compelling evidence. However, a paucity of knowledge exists regarding the barriers hindering effective T2T management in China. This study seeks to investigate the factors contributing to treatment failure within the context of the T2T strategy. A cross-sectional, multi-center investigation was conducted, involving the completion of electronic questionnaires by outpatients undergoing urate-lowering treatment for a duration exceeding 6 months. These questionnaires encompassed demographic information, disease-related conditions, comorbid conditions, and management. The study analyzed factors associated with serum uric acid levels exceeding 360 µmol/L, poor disease control, and poor medication adherence. A total of 425 valid questionnaires were collected, representing 90.8% of the patients. The T2T implementation rate was 26.82% (n = 114). Factors linked to serum uric acid levels surpassing 360 µmol/L included moderate medication adherence (odds ratio (OR) = 2.35; 95% confidence interval (CI) 1.17–4.77; *P* = .016), poor medication adherence (OR = 4.63; 95% CI 2.28–9.51; *P* < .001), and management by general practitioners (OR = 0.60; 95% CI 0.37–0.97; *P* = .036). The rate of well-controlled patients was 14.35% (n = 61). Predictors of not well controlled encompassed the presence of tophi (OR = 2.48; 95% CI 1.17–5.61; *P* = .023), general medication adherence (OR = 2.78; 95% CI 1.28–6.05; *P* = .009), poor medication adherence (OR = 6.23; 95% CI 2.68–14.77; *P* < .001), and poor patient’s perception of gout (OR = 4.07; 95% CI 1.41–13.91; *P* = .015). A poor medication adherence rate of 55.29% (n = 235) was observed, with lower rates of poor medication adherence associated with the use of febuxostat (OR = 0.35; 95% CI 0.14–0.83; *P* = .02), uric acid levels exceeding 360 µmol/L (OR = 3.05; 95% CI 1.84–5.12; *P* = .00), moderate patient education (OR = 2.28; 95% CI 1.29–4.15; *P* = .01), moderate diet control (OR = 1.98; 95% CI 1.17–3.41; *P* = .01), and poor diet control (OR = 3.73; 95% CI 1.26–12.83; *P* = .02). The rate of T2T implementation in China is notably low among patients undergoing urate-lowering treatment of gout beyond 6 months. Importantly, medication adherence demonstrates a significant association with T2T outcomes.

**Key points**

- The rate of T2T was low in gout patient.
- Medication adherence is a crucial determinant of T2T success.

## 1. Introduction

Gout, a prevalent arthritic condition, arises from the deposition of monosodium urate crystals. In the United States, gout affects approximately 3% to 4% of adults.^[1]^ The purpose of gout treatment is to quickly and effectively relieve and eliminate acute symptoms, prevent the recurrence of acute arthritis, and reduce serum uric acid (sUA).^[2]^ Nonsteroidal anti-inflammatory drugs are an important option in gout acute attacks, which can provide both anti-inflammation and analgesia.^[3–5]^ For patients with gout, urate-lowering treatment (ULT) is imperative, as it mitigates the frequency of gout attacks, reduces the involvement of joints, diminishes urate deposition, and safeguards articular cartilage and renal function.^[6]^ Notably, the American College of Rheumatology (ACR) released guidelines in 2020 advocating for the treat-to-target (T2T) strategy in gout treatment.^[7]^ These recent guidelines provide clinicians with directives on managing gout cases within the framework of a T2T approach. Specifically, the treatment guidelines endorse striving for a sUA target of <360 μmoI/L for all gout patients and <300 μmoI/L for those afflicted with tophaceous or severe gout.^[8,9]^ However, a prevalence study revealed that only 22.3% of patients reached the stipulated target level, with the rate dwindling to a mere 11% for those on ULT for more than 12 months.^[10]^

The European League Against Rheumatism guidelines emphasize the importance of patient education and lifestyle modifications as integral components of optimal long-term gout management.^[11]^ Despite these recommendations, adherence to long-term ULT remains suboptimal.^[12–14]^ The under-recognition of gout and the ensuing societal burden contribute to inadequately controlled sUA levels.^[15,16]^ Consequently, cultivating patients’ comprehension of the pivotal role of adhering to ULT and sustaining the targeted sUA level over the long term is imperative.^[17]^ While interventions led by nurses,^[18]^ pharmacists,^[19,20]^ and physicians^[7]^ to enhance T2T outcomes have been documented, other factors potentially influence the T2T strategy due to divergent medical policies across nations. Despite multiple studies shedding light on the impact of allopurinol on T2T, research focusing on the clinical uric acid-lowering effect of febuxostat remains limited. In recent times, increased attention towards T2T has surfaced in European and American countries, yet its exploration in Asia remains constrained.^[21]^ To date, Asia’s contribution to this realm primarily encompasses a single-center, short-term study in China,^[22]^ and a small-sample study in the Philippines.^[23]^ Given these gaps, we have embarked on a comprehensive, multi-center study on T2T. Our study aims to evaluate factors—such as demographic variables, disease-related conditions, comorbidities, and management approaches—that correlate with instances of failing to adhere to the T2T strategy in China.

## 2. Methods

### 2.1. Study design and setting

This cross-sectional study was conducted between July 2020 and May 2021 at 7 tertiary-level hospitals (chictr.org.cn ChiCTR2000034700). The study received approval from the Institutional Medical Ethics Committee of the Fourth Clinical Medical College of Guangzhou University of Chinese Medicine.

### 2.2. Participants

Informed written consent was procured from all participating individuals. The study enrolled male and female outpatients aged between 18 and 80 years. These patients fulfilled the criteria for acute gouty arthritis as approved by the ACR 1977^[24]^ or the ACR/European League Against Rheumatism gout classification of 2015. These criteria were based on the pattern of joint and bursa involvement during symptomatic episodes, serum urate levels, synovial fluid analysis from a symptomatic episode, and imaging evidence of urate deposition in affected joints or bursae.^[25]^ Rheumatologists recommended the participation of patients who had undergone ULT for more than 6 months, were capable of independently completing the questionnaire, and were not afflicted with secondary gout or malignant tumors.

The state of well-controlled disease was defined as having reached the treatment target and experiencing no flare-ups or use of anti-inflammatory medication for a month.^[26]^ A total of 468 gout patients were consecutively invited to partake, with 425 valid questionnaires collected for subsequent statistical analysis after excluding 43 patients who were ineligible, 10 patients who did not meet inclusion criteria, and 33 patients who met exclusion criteria.

### 2.3. Data sources/measurement

To ensure representativeness, each participating physician was limited to assessing 5 consecutive patients. Electronic questionnaires were utilized for data collection. Gout patients were required to complete a standardized set of self-report questionnaires. The questionnaires were completed under the supervision of the doctor. These encompassed demographic variables like age, gender, body mass index categories (general: 18.5–23.9, overweight: 24–27.9, obesity: >28), education level, nonmanual labor, and family history. Disease-related conditions included disease duration, ULT duration, presence of tophi, medication usage, and the 8-item Morisky Medication Adherence Scale (poor medication adherence = <6, moderate medication adherence = 6–8, good medication adherence = 8).^[27]^ Additionally, comorbid conditions encompassed hypertension, diabetes, hyperlipidemia, kidney stones, and coronary atherosclerotic heart disease.

Management factors encompassed patient education (Good: comprehensive >6, Moderate: general 4–6, Poor: lacking <4),^[18,28,29]^ diet control (Good: total purine intake over 2 days <1.3 g, Moderate: total purine intake over 2 days 1.3–2.28 g, Poor: total purine intake over 2 days >2.28 g),^[7,30]^ exercise (Good: 3 or more times a week, each session lasting over 30 minutes, Moderate: 1–2 times a week, Poor: <1 time a week),^[31]^ heightened patient awareness of gout (encompassing understanding of gout, lifestyle adjustments, and treatment adherence), and knowledge level of gout patients (Good: high >8, Moderate: general 5–8, Poor: lacking ＜5).^[32]^ Regular follow-up based on the clinic records (Good: punctual, Moderate: occasional, Poor: absent), patients mutual help organization (formation of self-management collectives among patients), nonmedical security, economic burden of patients (exceeding 10% of monthly income), establishment of health records (chronic disease clinical records), and management from general practitioners were considered in this investigation.

### 2.4. Bias

The study adopted a sampling survey approach rather than a census, which might have introduced selection bias. Efforts were made to ameliorate recall bias by documenting each patient’s case whenever feasible.

### 2.5. Study size

The sample size was determined through nQuery Advisor software (Statistical Solutions Company, Ireland). Drawing on data from the Chinese Rheumatism Data Center 2016, where the rate of achieving target sUA levels over a 6-month period was 38.20%, assuming a rate of 0.4 with an acceptable error of 0.05, the estimated sample size was 369.

### 2.6. Statistical methods

Continuous variables were described using the median (from the 25th percentile to the 75th percentile), while categorical variables were presented as frequencies and percentages. The characteristics of subjects within the sUA > 360 µmol/L and sUA ≤ 360 µmol/L groups were compared employing independent *t* tests for normally distributed data and Mann–Whitney *U* tests for non-normally distributed data. Univariate analysis was conducted to identify variables associated with sUA > 360 µmol/L and well-controlled levels. For the purpose of identifying potential risk factors linked to poor compliance, a univariate logistic regression was performed individually for each variable (e.g., demographics, disease-related conditions, comorbidities, disease management). The independent variables displaying statistical significance were incorporated into the subsequent multivariate logistic regression. To circumvent the potential destabilizing impact of multicollinearity in the multivariate logistic regression model, we calculated the generalized variance inflation factor (GVIF) for each independent variable, and retained only those GVIF values ^[1/(2×df)]^ below 1.54, signifying the absence of multicollinearity. Following this, we established a comprehensive model with the remaining variables and subsequently conducted a stepwise regression utilizing a backward selection approach to derive a simplified model with the lowest Akaike information criterion. Associations were conveyed as odds ratios (ORs) accompanied by their corresponding 95% confidence intervals (CIs). We assessed the performance of the nomogram in terms of discrimination and calibration. Specifically, we assessed its discrimination ability by estimating the area under the receiver operating characteristic curve. A bootstrapping approach with 1000 repetitions was employed to validate the model. All analyses were conducted exclusively on complete cases, without any imputation carried out. A *P* value of .05 denoted statistical significance. The entirety of statistical analyses was executed utilizing R version 4.0.2 (IDE-RStudio Company, America).

## 3. Results

### 3.1. Clinical characteristics associated with target sUA

Comparison of patient characteristics in gout patients was carried out between the sUA >360 µmol/L (n = 311, 73.18%) and ≤360 µmol/L (n = 114, 26.82%) groups, as presented in Table 1. Within the sUA > 360 µmol/L group, a larger proportion of patients (70.4%) possessed a higher level of education compared to the sUA ≤ 360 µmol/L group (59.6%). Notably, comorbidity with diabetes exhibited a statistically significant difference in the sUA ≤ 360 µmol/L group (1.6% vs 6.1%, *P* = .02). Significant variations were observed between the ≤360 µmol/L and sUA > 360 µmol/L groups in terms of medication adherence (good 7.4% vs 23.7%, general 30.5% vs 39.5%, poor 62.1% vs 36.8%, *P* < .001) and regular follow-up (good 54.3% vs 67.5%, general 26.4% vs 20.2%, poor 62.1% vs 36.8%, *P* = .014). Moreover, noteworthy distinctions were discerned concerning the economic burden on patients (32.8% vs 44.7%, *P* = .03) and management from general practitioners (32.5% vs 46.5%, *P* = .009) across the 2 groups.

**Table 1:** Characteristics of the patients with gout between the sUA > 360 µmol/L and ≤360 µmol/L.

Characteristics	Uric acid level		*P* value
	>360 µmol/L(N = 311)	≤360 µmol/L (N = 114)	
**Demographics**			
Age, yr	42.0 (33.0–50.0)	45.0 (37.0–51.0)	.057
Sex, male (%)	309/311 (99.4)	110/114 (96.5)	.047
BMI (%)			.090
General	71/311 (22.8)	31/114 (27.2)	
Overweight	147/311 (47.3)	59/114 (51.8)	
Obesity	93/311 (29.9)	24/114 (21.1)	
Education level, ≥15 yr (%)	219/311 (70.4)	68/114 (59.6)	.047
Mental labor (%)	244/311 (78.5)	84/114 (73.7)	.300
Family history (%)	82/311 (26.4)	37/114 (32.5)	.224
**Disease-related conditions**			
Disease duration, yr	6.0 (3.0–10.0)	5.0 (2.2–14.0)	.884
Duration of ULT, yr	3.0 (1.0–5.0)	2.0 (1.0–5.0)	.111
Tophi (%)	108/311 (34.7)	35/114 (30.7)	.488
Used medicine (%)			
Analgesics	151/311 (48.6)	47/114 (41.2)	.189
Allopurinol	31/305 (10.2)	14/110 (12.7)	.476
Febuxostat	281/305 (92.1)	98/110 (89.1)	.328
Benzbromarone	42/305 (13.8)	20/110 (18.2)	.277
Medication adherence (%)			<.001
Good	23/311 (7.4)	27/114 (23.7)	
Moderate	95/311 (30.5)	45/114 (39.5)	
Poor	193/311 (62.1)	42/114 (36.8)	
**Comorbid conditions** (%)			
Hypertension	62/311 (19.9)	33/114 (28.9)	.065
Diabetes	5/311 (1.6)	7/114 (6.1)	.020
Hyperlipidemia	94/311 (30.2)	35/114 (30.7)	1.000
Kidney stones	70/266 (26.3)	34/103 (33.0)	.200
Coronary heart disease	4/311 (1.3)	1/114 (0.9)	
**Management** (%)			
Patients’ education			.772
Good	243/311 (78.1)	91/114 (79.8)	
Moderate	65/311 (20.9)	20/114 (17.5)	
Poor	3/311 (1.0)	3/114 (2.6)	
Diet control			.060
Good	63/311 (20.3)	36/114 (31.6)	
Moderate	231/311 (74.3)	70/114 (61.4)	
Poor	17/311 (5.5)	8/114 (7.0)	
Exercise			.219
Good	83/311 (26.7)	34/114 (29.8)	
Moderate	76/311 (24.4)	33/114 (28.9)	
Poor	152/311 (48.9)	47/114 (41.2)	
Patient’s perception of gout			.101
Good	63/311 (20.3)	30/114 (26.3)	
Moderate	188/311 (60.5)	68/114 (59.6)	
Poor	60/311 (19.3)	16/114 (14.0)	
Regular follow-up			.014
Good	169/311 (54.3)	77/114 (67.5)	
Moderate	82/311 (26.4)	23/114 (20.2)	
Poor	60/311 (19.3)	14/114 (12.3)	
Patients’ mutual help organization	159/311 (51.1)	64/114 (56.1)	.382
Nonmedical insurance	128/311 (41.2)	52/114 (45.6)	.439
Economic burden of patients	102/311 (32.8)	51/114 (44.7)	.030
Established health record	94/311 (30.2)	44/114 (38.6)	.128
Management from general practitioners	101/311 (32.5)	53/114 (46.5)	.009

BMI = body mass index, sUA = serum uric acid, ULT = urate-lowering treatment.

### 3.2. The factors associated with sUA > 360 µmol/L for patients with gout

**Figure 1.**
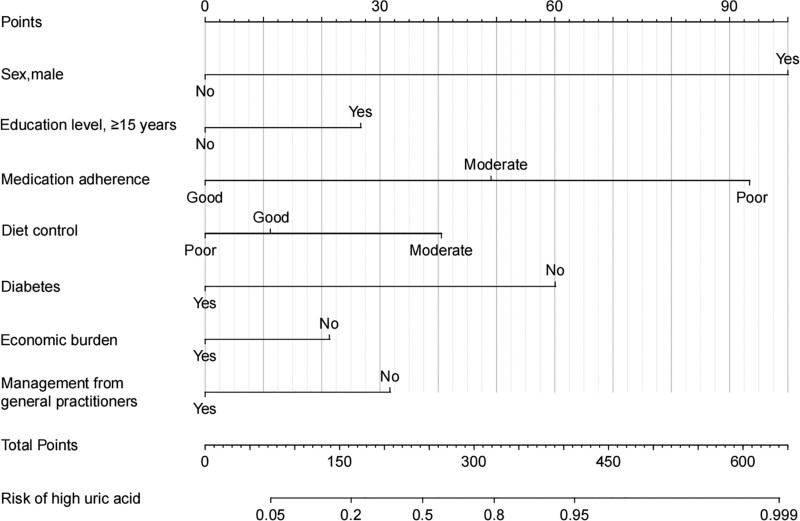
The nomogram illustrates the prediction model for high uric acid levels (>360 µmol/L) among patients with gout. The final model incorporates 7 covariates: gender, education, medication adherence, diet control, diabetes, economic burden, and management from general practitioners.

**Figure 2.**
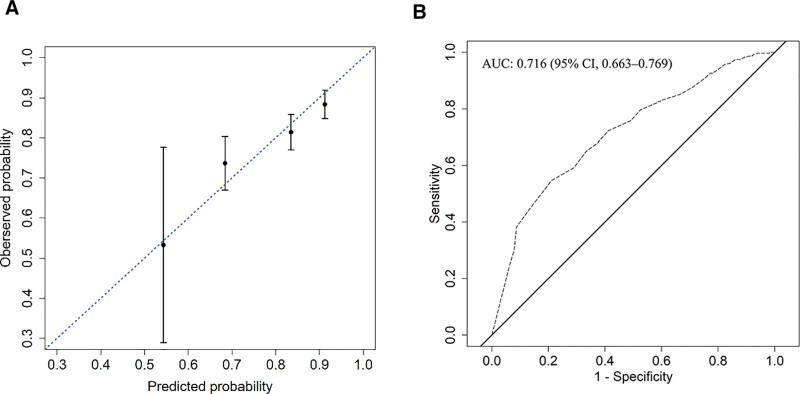
(A) The calibration plot showcases the agreement between predicted and observed probabilities, with the dashed line representing the ideal reference line. The points with error bars represent nomogram-predicted probabilities and corresponding 95% confidence intervals grouped across 4 quartile segments. (B) The ROC curve provides insight into the model’s predictive accuracy for high uric acid risk. ROC = receiver operating characteristic.

Subsequently, a multivariable logistic regression was employed to formulate the risk model and select factors, adhering to both statistical significance (*P* < .05) and clinical significance. We investigated factors linked to a sUA level exceeding 360 µmol/L among gout patients (Table 2). The findings revealed that management from general practitioners, general and poor medicine adherence were significantly associated with sUA levels > 360 µmol/L (*P* < .05). A nomogram predicting elevated sUA levels > 360 µmol/L in gout patients is illustrated in Figure 1. The nomogram had moderate discrimination with an area under the receiver operating characteristic curve of 0.716 (95% CI 0.663–0.769). To validate the model, a bootstrapping approach with 1000 repetitions was employed, yielding bias-corrected accuracy measures of a Brier score of 0.177, a calibration slope of 0.99, and a c-index of 0.726, as depicted in Figure 2.

**Table 2:** Results of multivariate logistic regression models to assess the factors associated with uric acid > 360 µmol/L for patients with gout.

Variables	OR (95% CI)	*P* value
Age, yr	1.00 (0.98–1.02)	.932
Sex, male	5.19 (0.89–41.98)	.079
BMI		
General	Ref	–
Overweight	1.11 (0.62–1.96)	.714
Obesity	1.62 (0.82–3.23)	.166
Education, university	1.62 (0.96–2.74)	.069
Regular follow-up		
Good	Ref	–
Moderate	1.24 (0.67–2.33)	.500
Poor	1.40 (0.68–3.01)	.367
Medication adherence		
Good	Ref	–
Moderate	2.35 (1.17–4.77)	.016
Poor	4.63 (2.28–9.51)	<.001
Diet control		
Good	Ref	–
Moderate	1.59 (0.92–2.74)	.095
Poor	0.71 (0.25–2.06)	.512
Diabetes	0.35 (0.09–1.32)	.124
Economic burden of patients	0.67 (0.41–1.10)	.109
Management from general practitioners	0.60 (0.37–0.97)	0.036

The sample size for fitting the full model and simplified model was 425.

BMI = body mass index, CI = confidence interval, OR = odds ratio.

### 3.3. Characteristics of not well-controlled and well-controlled sUA

Even upon achieving the target sUA level, certain patients persisted in experiencing joint symptoms. As a result, we conducted a further analysis to discern discrepancies between not well-controlled and well-controlled sUA levels. The particulars of patients are delineated in Table 3. The male patient ratio was notably higher in the not well-controlled group in contrast to the well-controlled group (99.2% vs 95.1%, *P* = .041). Substantive contrasts were identified in terms of regular follow-up (good 54.9% vs 75.4%, moderate 26.4% vs 14.8%, poor 18.7% vs 9.8%, *P* = .004), medication adherence (good 8.5% vs 31.1%, moderate 31.3% vs 42.6%, poor 60.2% vs 26.2%, *P* < .001), patients’ perception of gout (good 19.5% vs 36.1%, moderate 61.0% vs 55.7%, poor 19.5% vs 8.2%, *P* = .001), established health record (30.5% vs 44.3%, *P* = .039), and management from general practitioners (34.1% vs 49.2%, *P* = .030) between the not well-controlled and well-controlled groups.

**Table 3:** Characteristics of the patients with gout between the not well-controlled and well-controlled.

Characteristics	Not well controlled (N = 361)	Well controlled (N = 61)	*P* value
**Demographics**			
Age, yr	42.0 (33.0–50.0)	41.0 (35.0–51.0)	.813
Sex, male (%)	361/364 (99.2)	58/61 (95.1)	.041
BMI (%)			.052
General	81/364 (22.3)	21/61 (34.4)	
Overweight	179/364 (49.2)	27/61 (44.3)	
Obesity	104/364 (28.6)	13/61 (21.3)	
Education level, ≥15 yr (%)	244/364 (67.0)	43/61 (70.5)	.659
Mental labor (%)	278/364 (76.4)	50/61 (82.0)	.411
Family history (%)	103/364 (28.3)	16/61 (26.2)	.878
**Disease-related conditions**			
Disease duration, yr	6.0 (3.0–10.0)	5.0 (2.0–10.0)	.057
Duration of ULT, yr	3.0 (1.0–5.0)	2.0 (1.0–4.0)	.101
Tophi (%)	129/364 (35.4)	14/61 (23.0)	.058
Used medicine (%)			
Painkillers	198/364 (54.4)	0/61 (0.0)	
Allopurinol	42/355 (11.8)	3/60 (5.0)	.175
Febuxostat	326/355 (91.8)	53/60 (88.3)	.332
Benzbromarone	53/355 (14.9)	9/60 (15.0)	1.000
Regular follow-up (%)			.004
Good	200/364 (54.9)	46/61 (75.4)	
Moderate	96/364 (26.4)	9/61 (14.8)	
Poor	68/364 (18.7)	6/61 (9.8)	
Medication adherence (%)			<.001
Good	31/364 (8.5)	19/61 (31.1)	
Moderate	114/364 (31.3)	26/61 (42.6)	
Poor	219/364 (60.2)	16/61 (26.2)	
**Comorbid conditions** (%)			
Hypertension	80/364 (22.0)	15/61 (24.6)	.622
Diabetes	9/364 (2.5)	3/61 (4.9)	.392
Hyperlipidemia	109/364 (29.9)	20/61 (32.8)	.654
Kidney stones	87/316 (27.5)	17/53 (32.1)	.511
Coronary heart disease	5/364 (1.4)	0/61 (0.0)	
**Management (%**)			
Patients’ education			.095
Good	281/364 (77.2)	53/61 (86.9)	
Moderate	78/364 (21.4)	7/61 (11.5)	
Poor	5/364 (1.4)	1/61 (1.6)	
Diet control			.247
Good	82/364 (22.5)	17/61 (27.9)	
Moderate	259/364 (71.2)	42/61 (68.9)	
Poor	23/364 (6.3)	2/61 (3.3)	
Exercise			.086
Good	98/364 (26.9)	19/61 (31.1)	
Moderate	88/364 (24.2)	21/61 (34.4)	
Poor	178/364 (48.9)	21/61 (34.4)	
Patient’s perception of gout			.001
Good	71/364 (19.5)	22/61 (36.1)	
Moderate	222/364 (61.0)	34/61 (55.7)	
Poor	71/364 (19.5)	5/61 (8.2)	
Patients’ mutual help organization	189/364 (51.9)	34/61 (55.7)	.678
Nonmedical security	154/364 (42.3)	26/61 (42.6)	1.000
Economic burden of patients	126/364 (34.6)	27/61 (44.3)	.152
Established health record	111/364 (30.5)	27/61 (44.3)	.039
Management from general practitioners	124/364 (34.1)	30/61 (49.2)	.030

BMI = body mass index, ULT = urate-lowering treatment.

### 3.4. Nomogram prediction of not well-controlled sUA

**Figure 3.**
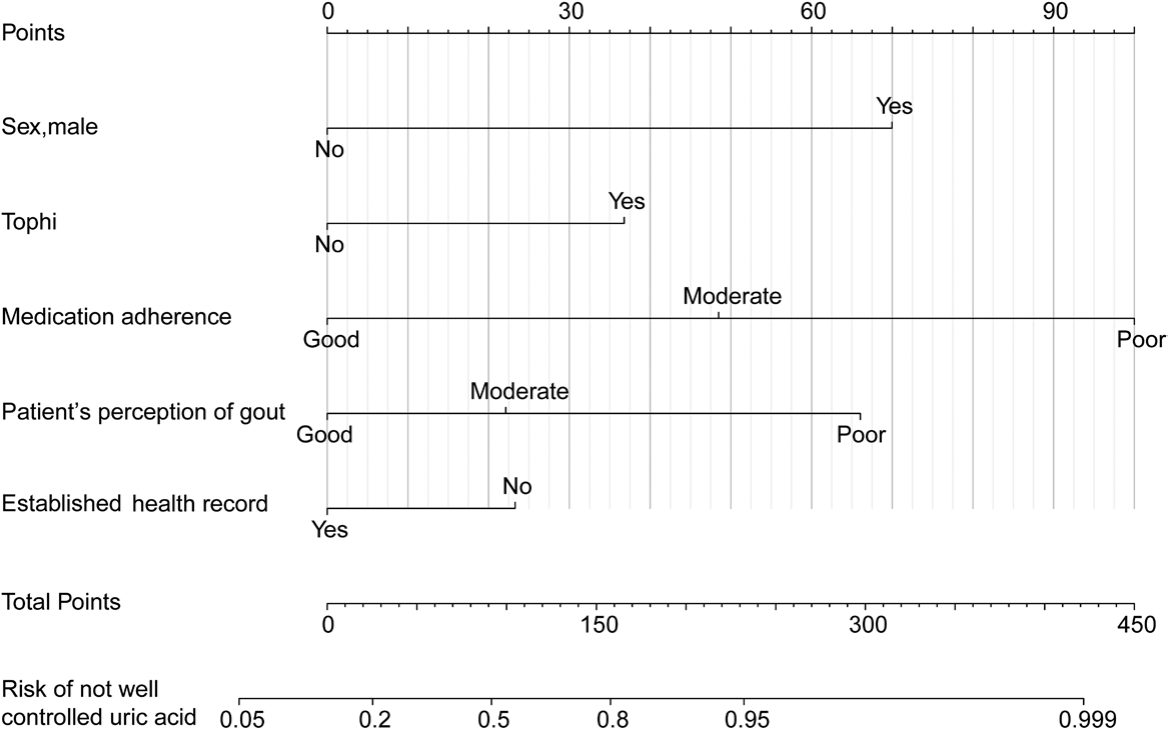
The nomogram depicts the prediction model for the risk of not well-controlled uric acid levels among patients with gout. The final model integrates 5 covariates: gender, presence of tophi, medication adherence, patient’s perception of gout, and the presence of established health records.

Predictors of not well-controlled sUA were evaluated using multivariate logistic regression models (Table 4). Our findings demonstrated that the presence of tophi (OR = 2.48; 95% CI 1.17–5.61; *P* = .023), poor patient’s perception of gout (OR = 4.07; 95% CI 1.41–13.91; *P* = .015), moderate medication adherence (OR = 2.78; 95% CI 1.28–6.05; *P* = .009), and poor medication adherence (OR = 6.23; 95% CI 2.68–14.77; *P* < .001) were associated with an elevated risk of not well-controlled sUA. The nomogram designed to predict not well-controlled sUA in gout patients is depicted in Figure 3. The GVIF for the potential predictors of well-controlled sUA in patients with gout is illustrated in Table 5.

**Table 4:** Results of multivariate logistic regression models to assess predictors of not well-controlled for patients with gout.

Variables	Full model		Simplified model	
	OR (95% CI)	*P* value	OR (95% CI)	*P* value
Age, yr	1.00 (0.97–1.03)	.956	–	–
Sex, male	5.79 (0.83–40.66)	.068	4.32 (0.68–27.69)	.109
BMI				
General	Ref	–	–	–
Overweight	1.58 (0.77–3.22)	.205	–	–
Obesity	1.58 (0.68–3.76)	.292	–	–
Disease duration, yr	0.96 (0.91–1.02)	.151	–	–
Tophi	2.48 (1.17–5.61)	.023	2.16 (1.11–4.44)	.028
Regular follow-up				
Good	Ref	–	–	–
Moderate	1.70 (0.73–4.35)	.242	–	–
Poor	1.42 (0.55–4.21)	.491	–	–
Medication adherence				
Good	Ref	–	Ref	–
Moderate	2.78 (1.28–6.05)	.009	2.76 (1.30–5.87)	.008
Poor	6.23 (2.68–14.77)	<.001	8.10 (3.66–18.32)	<.001
Patients’ education				
Good	Ref	–	–	–
Moderate	1.60 (0.67–4.35)	.316	–	–
Poor	1.54 (0.12–45.03)	.763	–	–
Exercise				
Good	Ref	–	–	–
Moderate	0.71 (0.33–1.52)	.375	–	–
Poor	1.48 (0.69–3.18)	.309	–	–
Patient’s perception of gout				
Good	Ref	–	Ref	–
Moderate	1.67 (0.82–3.36)	.154	1.59 (0.81–3.06)	.169
Poor	4.07 (1.41–13.91)	.015	3.98 (1.45–12.97)	.012
Established health record	0.77 (0.35–1.68)	.502	0.61 (0.34–1.13)	.115
Management from general practitioners	0.72 (0.33–1.56)	96	–	–

The sample size for fitting the full model and simplified model was 425.

BMI = body mass index, CI = confidence interval, OR = odds ratio.

**Table 5:** Generalized variance inflation factors (GVIF) for candidate predictors of high uric acid > 360 µmol/L and well-controlled among patients with gout.

Variables	GVIF of uric acid level model	GVIF of well controlled model
Age, yr	1.14	1.21
Sex, vs female	1.02	1.04
BMI, vs general	1.03	1.05
Education level, ≥15 yr	1.09	–
Regular follow-up	1.07	1.07
Medication adherence, vs good	1.05	1.05
Diet control, vs good	1.04	–
Diabetes	1.03	–
Economic burden	1.04	–
Management from general practitioners	1.03	1.30
Disease duration, yr	–	1.31
Tophi	–	1.14
Patients’ education, vs good	–	1.05
Exercise, vs good	–	1.04
Patient’s perception of gout, vs good	–	1.05
Established health record	–	1.29

BMI = body mass index, GVIF = generalized variance inflation factors.

### 3.5. Characteristics of medication adherence for patients with gout

Medication adherence exhibited a robust association with the T2T approach. To delve further, subgroup analyses were conducted, comparing patient characteristics within the poor medication adherence group and the combined good + moderate medication adherence group (Table 6). Noteworthy findings emerged: medication utilization of analgesics (poor 57.0% vs good + moderate 33.7%; OR = 2.61; 95% CI 1.76–3.90; *P* = .00), allopurinol (poor 13.6% vs good + moderate 7.5%; OR = 1.94; 95% CI 1.02–3.88; *P* = .05), and febuxostat (poor 88.2% vs good + moderate 95.2%; OR = 0.38; 95% CI 0.16–0.79; *P* = .01) displayed associations with medication adherence. Notably, uric acid > 360 µmol/L (poor 82.1% vs good + moderate 62.1%; OR = 2.80; 95% CI 1.81–4.40; *P* = .00) and hyperlipidemia (poor 34.9% vs good + moderate 24.7%; OR = 1.63; 95% CI 1.07–2.51; *P* = .02) underscored the statistical disparities between the poor and good + moderate medication adherence groups. Furthermore, statistical differences were evident in patients’ education (moderate 26.8% vs 11.6%; OR = 2.83; 95% CI 1.69–4.90; *P* = .00), diet control (moderate 74.9% vs 65.8%, OR = 2.08, 95% CI 1.31–3.31, *P* = .00; poor 8.1% vs 3.2%, OR = 4.67, 95% CI 1.80–13.77, *P* = .00), exercise (poor 52.3% vs 40.0%, OR = 1.97, 95% CI 1.22–3.20, *P* = .02), high attention of patients to gout (poor 62.1% vs good + moderate 75.8%; OR = 0.52; 95% CI 0.34–0.80; *P* = .00), and patients’ perception of gout (moderate 65.1% vs 54.2%; OR = 1.76; 95% CI 1.11–2.80; *P* = .01) within the poor and good + moderate medication adherence groups.

**Table 6:** Descriptive statistics and univariate logistic regression model of potential factors associated with poor medication adherence for patients with gout.

Characteristics	Medication adherence		Unadjusted analysis	
	Poor (n = 235)	Good + Moderate (n = 190)	OR (95% CI)	*P* value
**Demographics**				
Age, yr	42.0 (34.0–50.0)	43.0 (34.0–50.8)	0.99 (0.98–1.01)	.43
Sex, male (%)	233/235 (99.1)	186/190 (97.9)	2.51 (0.48–18.21)	.29
BMI, kg/m^2^	26.0 (24.2–28.3)	25.7 (23.8–28.1)	1.04 (0.99–1.10)	.12
BMI (%)				
General	48/235 (20.4)	54/190 (28.4)	1 (ref)	
Overweight	120/235 (51.1)	86/190 (45.3)	1.57 (0.97–2.54)	.06
Obesity	67/235 (28.5)	50/190 (26.3)	1.51 (0.88–2.58)	.13
Education level, ≥15 yr (%)	156/235 (66.4)	131/190 (68.9)	0.89 (0.59–1.34)	.57
Mental labor (%)	181/235 (77.0)	147/190 (77.4)	0.98 (0.62–1.54)	.93
Family history (%)	70/235 (29.8)	49/190 (25.8)	1.22 (0.80–1.88)	.36
**Disease-related conditions**				
Disease duration, yr	6.0 (3.5–10.0)	5.0 (3.0–10.0)	1.00 (0.97–1.03)	.96
Duration of ULT, yr	3.0 (1.0–5.0)	2.0 (1.0–5.0)	1.05 (0.99–1.11)	.08
Tophi (%)	82/235 (34.9)	61/190 (32.1)	1.13 (0.76–1.70)	.55
Used medicine (%)				
Analgesics	134/235 (57.0)	64/190 (33.7)	2.61 (1.76–3.90)	.00
Allopurinol	31/228 (13.6)	14/187 (7.5)	1.94 (1.02–3.88)	.05
Febuxostat	201/228 (88.2)	178/187 (95.2)	0.38 (0.16–0.79)	.01
Benzbromarone	39/228 (17.1)	23/187 (12.3)	1.47 (0.85–2.60)	.17
Uric acid, >360 µmol/L (%)	193/235 (82.1)	118/190 (62.1)	2.80 (1.81–4.40)	.00
**Comorbid conditions** (%)				
Hypertension	48/235 (20.4)	47/190 (24.7)	0.78 (0.49–1.24)	.29
Diabetes	5/235 (2.1)	7/190 (3.7)	0.57 (0.17–1.81)	.34
Hyperlipidemia	82/235 (34.9)	47/190 (24.7)	1.63 (1.07–2.51)	.02
Kidney stones	57/204 (27.9)	47/165 (28.5)	0.97 (0.62–1.54)	.91
Coronary heart disease	4/235 (1.7)	1/190 (0.5)	3.27 (0.48–64.31)	.29
**Management** (%)				
Patients’ education				
Good	168/235 (71.5)	166/190 (87.4)	1 (ref)	
Moderate	63/235 (26.8)	22/190 (11.6)	2.83 (1.69–4.90)	.00
Poor	4/235 (1.7)	2/190 (1.1)	1.98 (0.38–14.39)	.44
Diet control				
Good	40/235 (17.0)	59/190 (31.1)	1 (ref)	
Moderate	176/235 (74.9)	125/190 (65.8)	2.08 (1.31–3.31)	.00
Poor	19/235 (8.1)	6/190 (3.2)	4.67 (1.80–13.77)	.00
Exercise				
Good	56/235 (23.8)	61/190 (32.1)	1 (ref)	
Moderate	56/235 (23.8)	53/190 (27.9)	1.15 (0.68–1.94)	.60
Poor	123/235 (52.3)	76/190 (40.0)	1.76 (1.11–2.80)	.02
High attention of patients to gout	146/235 (62.1)	144/190 (75.8)	0.52 (0.34–0.80)	.00
Patient’s perception of gout				
Good	40/235 (17.0)	53/190 (27.9)	1 (ref)	
Moderate	153/235 (65.1)	103/190 (54.2)	1.97 (1.22–3.20)	.01
Poor	42/235 (17.9)	34/190 (17.9)	1.64 (0.89–3.03)	.11
Patients’ mutual help organization	131/235 (55.7)	92/190 (48.4)	1.34 (0.91–1.97)	.13
Nonmedical insurance	99/235 (42.1)	81/190 (42.6)	0.98 (0.67–1.44)	.92
Economic burden of patients	83/235 (35.3)	70/190 (36.8)	0.94 (0.63–1.40)	.75
Established health record	69/235 (29.4)	69/190 (36.3)	0.73 (0.48–1.10)	.13
Management from general practitioners	82/235 (34.9)	72/190 (37.9)	0.88 (0.59–1.31)	.52

BMI = body mass index, CI = confidence interval, OR = odds ratio, ULT = urate-lowering treatment.

### 3.6. The factors associated with poor medication adherence for patients with gout

Multivariate logistic regression models were utilized to evaluate factors associated with poor medication adherence in gout patients (Table 7). Our findings indicated that febuxostat utilization, uric acid >360 µmol/L, moderate patients’ education, and moderate to poor diet control were linked with poor medication adherence in gout patients.

**Table 7:** Results of multivariate logistic regression models to assess factors associated with poor medication adherence for patients with gout.

Characteristics	Full model		Simplified model	
	OR (95% CI)	*P* value	OR (95% CI)	*P* value
BMI				
General	1 (ref)		–	–
Overweight	1.35 (0.78–2.34)	.28	–	–
Obesity	0.99 (0.53–1.85)	.97	–	–
Gout flares in a month	1.33 (0.69–2.56)	.39	–	–
Used medicine				
Analgesics	1.71 (0.90–3.29)	.11	2.23 (1.45–3.46)	.00
Allopurinol	1.18 (0.54–2.66)	.68	–	–
Febuxostat	0.35 (0.14–0.83)	.02	0.33 (0.13–0.76)	.01
Uric acid, >360 µmol/L	3.05 (1.84–5.12)	.00	3.07 (1.87–5.10)	.00
Hyperlipidemia	1.29 (0.79–2.12)	.32	–	–
Patients’ education				
Good	1 (ref)		1 (ref)	
Moderate	2.28 (1.29–4.15)	.01	2.42 (1.38–4.35)	.00
Poor	2.49 (0.40–21.55)	.35	2.98 (0.51–24.46)	.25
Diet control				
Good	1 (ref)		1 (ref)	
Moderate	1.98 (1.17–3.41)	.01	1.93 (1.15–3.24)	.01
Poor	3.73 (1.26–12.83)	.02	4.45 (1.53–15.07)	.01
Exercise				
Good	1 (ref)		–	–
Moderate	1.04 (0.57–1.89)	.90	–	–
Poor	1.51 (0.89–2.58)	.13	–	–
High attention of patients to gout	0.73 (0.45–1.18)	.20	0.70 (0.44–1.13)	.14
Patient’s perception of gout				
Good	1 (ref)		–	–
Moderate	1.37 (0.79–2.39)	.26	–	–
Poor	1.12 (0.56–2.26)	.74	–	–

BMI = body mass index, CI = confidence interval, OR = odds ratio.

## 4. Discussion

In this study, the results obtained underscored a notable low rate of achieving T2T among patients undergoing ULT for more than 6 months. The enduring maintenance of the target sUA level over the long term emerged as a challenging pursuit. In alignment with established epidemiological trends, our study revealed a predilection for gout susceptibility among males, particularly those aged over 40 years.^[33]^ The factors contributing to the inability to attain the target sUA level predominantly encompassed the economic burdens borne by patients, irregular medical visits, and the lack of guidance from general practitioners. Intriguingly, our findings unveiled that a higher educational level in some patients correlated with failure to achieve the desired sUA levels, possibly attributed to lifestyle facets like dietary habits and demanding work commitments.

Furthermore, the documented significance of poor medication adherence as a gout risk factor accentuates its potential to lead to diminished T2T rates. Our investigations illuminated the pivotal role of medication adherence in the context of gout’s T2T paradigm. As is often observed in chronic ailments, adherence to long-term gout treatment regimens remained suboptimal. Specifically, merely 26.82% of our participants exhibited a commendable level of adherence to ULT, aligning with analogous findings from various Western gout studies. However, the general picture of ULT adherence among gout patients remained unsatisfactory.^[13]^ A comprehensive meta-analysis highlighted treatment adherence percentages spanning from 10% to 46%.^[34]^ Our study, in particular, highlighted a higher prevalence of poor medication adherence within the sUA > 360 µmol/L group. Notably, the deficiency in medication adherence resonated with aspects like patients’ education and diet control.^[35]^ Equally striking was the consistent observation that older age and the presence of comorbidities such as hypertension or diabetes were linked with improved adherence.^[13,34]^ In alignment with these findings, our study indicated inferior adherence among gout patients with concurrent hyperlipidemia. Conversely, another study illuminated that advanced age, higher body weight, the use of anti-hypertensive or colchicine medications, and the presence of conditions like dementia, diabetes, or dyslipidemia reduced the risk of non-persistence and nonadherence.^[36]^

Greater attention should be directed toward the management of chronic diseases to enhance medication adherence. Insufficient patient education can directly lead to a reduction in medication adherence.^[35]^ Our study underscores that the absence of management from general practitioners constitutes a risk factor. Additionally, limited knowledge about gout contributes to patients’ noncompliance with treatment regimens. The constraints on doctors’ time often hinder the provision of thorough education on gout and ULT to their patients, consequently leading to acute gout flares and a lack of sustained adherence to ULT for effective, long-term sUA level control.^[15]^ A minority of patients have been systematically educated on risk reduction strategies, addressing comorbidities, or personalized lifestyle recommendations pertinent to chronic gout.^[12,14]^ While interventions led by nurses^[18]^ and pharmacists^[19,20]^ to enhance T2T have been documented, the panel acknowledges the pivotal role of physicians in educating patients and implementing a T2T framework.^[7]^ Clinically, education concerning gout and its management principles forms the cornerstone of successful treatment. Encouraging a healthy lifestyle consistently and providing patients with pertinent disease management information are vital to fostering self-management and sustained adherence. Thus, bolstering health education becomes imperative for gout patients subject to recurrent and long-term ULT, with the ultimate goal of eradicating the root causes of gout pain and disease progression.^[37]^

The present study is not without its limitations. First, the scope of the sampled population and research centers may be considered somewhat limited. While this was a multi-center study, potential variations stemming from disparities between developed and underdeveloped regions, diverse Eastern and Western locales in China, and disparate dietary habits, might necessitate a more expansive sample size for conclusive validation. Second, our study exclusively delved into compliance rates exceeding 6 months. Hence, it accentuates the requirement for research spanning a minimum of 1 year’s adherence to provide a substantial foundation for future clinical interventions. Finally, given the cross-sectional nature of this study, our findings underscore the critical importance of physician-guided chronic disease management.

## 5. Conclusions

In summary, our study underscores that patients with gout undergoing ULT for over 6 months exhibit a notably low T2T rate in China. Furthermore, medication adherence plays a pivotal role in achieving T2T.

## Author contributions

**Data curation:** Sihui He, Zhiying Zhan.

**Funding acquisition:** Xia Qiu.

**Investigation:** Xueming Yao, Li Zhong, Jiaxin Wei.

**Project administration:** Ertao Jia.

**Writing – original draft:** Shasha Hu, Jianyong Zhang, Wukai Ma, Hongling Geng, Ertao Jia.
